# Metagenomic Approach Reveals Variation of Microbes with Arsenic and Antimony Metabolism Genes from Highly Contaminated Soil

**DOI:** 10.1371/journal.pone.0108185

**Published:** 2014-10-09

**Authors:** Jinming Luo, Yaohui Bai, Jinsong Liang, Jiuhui Qu

**Affiliations:** 1 Key Laboratory of Drinking Water Science and Technology, Research Center for Eco-Environmental Sciences, Chinese Academy of Sciences, Beijing, People's Republic of China; 2 University of Chinese Academy of Sciences, Beijing, People's Republic of China; Hospital for Sick Children, Canada

## Abstract

Microbes have great potential for arsenic (As) and antimony (Sb) bioremediation in heavily contaminated soil because they have the ability to biotransform As and Sb to species that have less toxicity or are more easily removed. In this study, we integrated a metagenomic method with physicochemical characterization to elucidate the composition of microbial community and functional genes (related to As and Sb) in a high As (range from 34.11 to 821.23 mg kg^−1^) and Sb (range from 226.67 to 3923.07 mg kg^−1^) contaminated mine field. Metagenomic analysis revealed that microbes from 18 phyla were present in the 5 samples of soil contaminated with high As and Sb. Moreover, redundancy analysis (RDA) of the relationship between the 18 phyla and the concentration of As and Sb demonstrated that 5 phyla of microbes, i.e. *Actinobacteria*, *Firmicutes*, *Nitrospirae*, *Tenericutes* and *Gemmatimonadetes* were positively correlated with As and Sb concentration. The distribution, diversity and abundance of functional genes (including *arsC*, *arrA*, *aioA*, *arsB* and ACR3) were much higher for the samples containing higher As and Sb concentrations. Based on correlation analysis, the results showed a positive relationship between *arsC*-like (R^2^ = 0.871) and *aioA*-like (R^2^ = 0.675) gene abundance and As concentration, and indicated that intracellular As(V) reduction and As(III) oxidation could be the dominant As detoxification mechanism enabling the microbes to survive in the environment. This study provides a direct and reliable reference on the diversity of microbial community and functional genes in an extremely high concentration As- and Sb-contaminated environment.

## Introduction

Arsenic (As) and antimony (Sb) are naturally occurring toxic elements, which are released into the natural environment either by natural activities or anthropogenic sources from various industries [1]. Both As and Sb can be strongly retained in soils [2], and their toxicity, behavior and bioavailability in the environment intensively rely on speciation and environmental conditions. Because of their similar chemistry, As and Sb are commonly associated with each other in the environment [3]. Despite their toxicity, functional microbes have still been reported to have survived in As- and Sb-contaminated environments [4], [5], which has implications for As and Sb bioremediation.

As and Sb microbial metabolisms include arsenate (As(V)) reduction [6], arsenite (As(III)) oxidation [6], As methylation [7], Sb(V) reduction [8], Sb(III) oxidation [5], and Sb methylation [7]. The biogeochemical cycling of As and Sb has been shown to be promoted based on redox reactions of microbes [9]. As we know, 99% of microorganisms in the natural environment have not been cultured. Traditional fingerprint methods, such as T-RFLP [10], DGGE and TGGE [11], can identify designated DNA fragments and analyze the composition of a microbial community efficiently, whereas these techniques still have some limitations and deficiencies. T-RFLP can only roughly analyze the microbial composition, and has difficulty identifying the species present. As for DGGE/TGGE, it can only analyze DNA fragments that are below 500 base pairs. Assessment of the composition of the whole community gene pool of microbial communities is limited using these methods.

Currently the metagenomic method is regarded as the most efficient, reliable, rapid and accurate way to reveal the entire microbial composition and genetic content of a community under complex environment conditions. It can identify the functional genes related to the metabolism of As and Sb, in order to provide reliable data on the phylogenetic composition and microbial metabolism synchronously [12]. A deep and direct insight into the soil biodiversity and microbial community and its functions can be investigated by using this novel molecular technique. Until now a few reports have been put forward studying water [13], sediment [14] and activated sludge [15] contaminated environments with relatively low concentrations of As by the metagenomic method. However, there are large numbers of As- and Sb-contaminated mine areas with high concentrations of As and Sb worldwide. And As and Sb are always co-exists in the natural environment. However, to the best of our knowledge, there are no reports analyzing As- and Sb-contaminated environments using the metagenomic method. Therefore, it is essential to gain a deep perspective and understanding of the diversity of microbes and functional genes under high concentrations of As and Sb, as well as co-existence of As and Sb through the metagenomic method.

In this study, we adopt a multidisciplinary approach to identify the inorganic components of the soil samples, and use a metagenomic-based molecular technique to decipher the distribution, diversity, and abundance of microbial communities in As- and Sb-contaminated environmental soil samples. The objectives of the current study are to (i) elucidate the whole microbial communities' composition and structure in soil contaminated by high As and Sb concentrations, (ii) identify the bacterium that plays a dominant role under different As and Sb contamination levels, (iii) investigate the abundance, distribution and diversity of functional genes related to As and Sb species transformations, and (iv) use statistical analysis methods to identify the relationship among microbes, redox genes' abundance and soil physicochemical parameters.

## Materials and Methods

### Ethics Statement

No specific permits were required for the described field studies in Lengshuijiang City, Hunan Province, China. The location is not privately-owned or protected in any way and the field studies did not involve endangered or protected species.

### Soil Sample Collection and Preparation

Five soil samples (named LSJ-1, LSJ-2, LSJ-3, LSJ-4 and LSJ-5) were collected from an As- and Sb-contaminated mine field in Lengshuijiang City, Hunan Province, China (**Table S1**). Soil samples were collected from 2–5 cm below the surface. The ambient temperature was about 15°C during sample collection. A portable freezer was used to keep the temperature at minus fifteen centigrade during sample collection. Soil samples were dried, pulverized, and sieved to 100 meshes before analysis of physicochemical properties.

### Physicochemical Analysis

The sieved soil samples were freeze-dried by a freeze drier (LGJ-10B, China) before analysis. pH values were measured by pH meter (Thermo Orion 3 STAR, USA) in a soil sample to water ratio of 1∶5 (w/w) after shaking about 24 h. The basic elements C, H and N were analyzed with an elemental analyzer (Elementar Analysen system GmbH, Germany). Before analyzing the total Sb, As, Pb, Cu and Mg, the soil samples were dissolved by a Microwave Reaction System (Multwave 3000) and then measured with an Atomic Absorption Spectrophotometer (ContrAA 700, Germany). Fourier transform infrared (FT-IR) spectroscopy was used to identify the organic compounds present in soil samples. X-ray photoelectron spectroscopy (XPS) was used to determine the distribution of elements.

### Soil DNA Extraction

Soil DNA was extracted from the contaminated field soil using the PowerSoil DNA Isolation Kit according to the manufacturer's instructions (Mobio, USA). A 0.25 g soil sample was added to each tube with three replicates, and the DNA was dissolved in 100 µL sterilized deionized water and stored at −20°C before use. The concentration of extracted nucleic acids was determined photometrically using an Eppendorf BioPhotometer plus (Hamburg, Germany).

### Real-time PCR

Real-time polymerase chain reaction (qPCR) was used to determine the quantitative distribution of As- and Sb-related genes and 16S rRNA bacteria. As(V)-reducing functional genes *arsC* (representing As-reducing bacteria under aerobic conditions) [16], *arrA* (representing As-reducing bacteria under anaerobic conditions) [17], As methylation functional gene *arsM* [18], As(III) oxidation gene *aroA* [19], as well as As(III) and Sb transfer gene *arsB* [20] were quantified using the SYBR Green I method [21]. The information about primer pairs and the PCR thermal programs is described in **Table S2**.

The SYBR Green I PCR reaction mixture includes 1 µL template (0.4 µL for each primers), 0.4 µL ROX Reference Dye II (50×), and 10 µL 2×SYBR Premix Ex Taq (Tli- RnaseH Plus) within 6.8 µL ddH_2_O in a 20 µL reaction. Reactions were performed on an ABI 7300 fast Real-time PCR system. Melting curves were performed for each reaction to confirm the purity of amplified products. Questionable products were verified on a 1.2% agarose gel.

Calibration curves were created for each sample by tenfold serial dilution of plasmids whose gene concentrations were quantified in triplicate. A standard curve of eight dilutions run in duplicate was generated for each qPCR plate. All DNA samples concentrations were measured with an Eppendorf BioPhotometer plus (Hamburg, Germany) immediately before diluting tenfold for standard curves. The concentrations of gene copies per gram of the soil were calculated from the gene copies detected in each microliter of DNA, multiplied by the dilution ratio of the DNA template as well as the volume of DNA extracted from one gram of original soil.

### Metagenomic Analysis

Metagenomic analysis was carried out to identify the composition and diversity of the microbial community in the soil.

DNA samples of the five soils were sent to BGI Company for sequencing. An Illumina TruSeq DNA Sample Preparation Kit (Illumina, Inc., San Diego, USA) was used to dispose metagenomic samples and construct two paired-end libraries (350 bp insert size). The prepared templates were measured on the Illumina TruSeq 2000 sequencing system for analysis (90 bp pair-end reads). Sequence reads were filtered, in order to remove adapters, low quality reads and those belonging to the host. We used a velvet *de novo* assembler [22] to assemble the filtered reads (∼2 Gbp for both datasets) into long contigs, and they were calculated on the cloud computing platform of the Beijing Computing Center (Beijing, China). MG-RAST on-line software [23] (http://metagenomics.anl.gov) was used to analyze the assembled contigs in order to investigate the taxonomic classification and function annotation.

Since no As or Sb metabolism genes or protein databases have been reported, we used the same subdatabases of *arrA, arsM and aioA* which have been put forward by Cai et al. [15] to analyze *arrA*, *arsM* and *aioA* in our samples. For *arsC*, ACR3 and *arsB* analysis, the subdatabases of *arsC*, ACR3 and *arsB* were built (**Table S3**). In order to analyze the distribution, diversity, and abundance of functional gene groups, we compared the original datasets with these subdatabases, and displayed it in a heatmap.

Five original metagenomic datasets were archived at the NCBI Sequence Read Archive (SRA) under the accession number of PRJNA239941.

### Statistical Analysis

To compare the community composition and function among the five samples, we used the statistical analysis of metagenomic profiles bioinformation software [24]. The two-sided Fisher's exact test was used to calculate the statistical significance, and the Newcombe-Wilson method with 95% confidence interval was used to analyze the differences between the proportions. The relationships between soil microbes and physicochemical parameters were examined by means of RDA applied to the correlation matrix of these variables. The analysis was performed in Canoco 4.5 for Windows [25]. SPSS 18.0 software was used to analyze the significant differences (P≤0.05) and the least significant difference (LSD) test after one-way analysis of variance (ANOVA) among five soil samples in Real-time PCR analysis. The other statistical analyses were carried out through OriginPro 8.5 software.

## Results and Discussion

### The Distribution of Inorganic Components in the Soil

**Figure 1:**
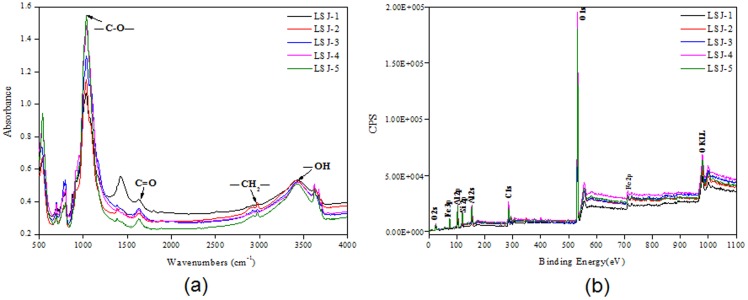
Chemical composition analysis of the soil samples were based on (a) FT-IR, (b) XPS.

The main physicochemical characteristics of the soil samples are shown in **Table 1**, which included the pH values, the contents of main elements C, N and H and the concentrations of Sb, As, and other heavy metals (including Pb, Cu, Cd, Cr, Fe, and Mg). In this study, the concentrations of As and Sb were much higher than those reported in the literature (**Table S4**). The five soil samples possessed similar pH values (pH 7.19 to 7.75), which could be due to the high buffering capacity of the clay soils. Even though the concentrations of As and Sb were extremely high in these environments, they fluctuated among the five samples. For LSJ-4 and LSJ-5, despite the close location of the two sites, the concentrations of As and Sb in LSJ-4 were much higher than in LSJ-5. Being aware that the soil sample of LSJ-5 was close to the root of plants while LSJ-4 was far from the root of plants, the discrepancy possibly arose because the hyperaccumulating plants [26] could adsorb As and Sb from the soil, and contributed to the lower As and Sb concentrations in LSJ-5. FT-IR and XPS data were analyzed in order to gain a further understanding of the physicochemical properties of the soil samples that could contribute to the composition and function of the microbial community. As shown in **Figure. 1a**, the FT-IR spectra reveal that the dominant organic functional groups in the soil samples were -CO-, C = O (NH_2_- and -NH-), CH_2_-, and OH- as recognized by peaks at around 1050 cm^−1^, 1700 cm^−1^, 2970 cm^−1^, 3420 cm^−1^, respectively [27]. XPS analysis identified that there was Fe, O, C, Si and Al in the soil samples (**Figure. 1b**). In summary, the results revealed that the concentrations of As and Sb varied among the five samples but the organic and inorganic compositions in the samples were identical.

**Table 1:** Physicochemical analysis of soil samples.

Sample	pH	Sb(mg/kg)	As(mg/kg)	Pb(mg/kg)	Cu(mg/kg)	Cd(mg/kg)	Cr(mg/kg)	Mg(mg/kg)	C(%)	N(%)	H(%)	Site
LSJ-1	7.75	355.41	34.11	19.58	23.71	—	—	2111.46	3.12	0.13	0.59	27°47′17′′N,111°27′46′′E
LSJ-2	7.64	1586.21	821.23	3.37	22.12	—	—	612.57	2.54	0.13	0.68	27°47′08′′N,111°29′35′′E
LSJ-3	7.19	3923.07	610.52	56.61	25.93	—	—	372.79	2.68	0.15	0.76	27°45′56′′N,111°29′11′′E
LSJ-4	7.32	1114.26	409.76	29.55	24.36	—	—	140.42	2.39	0.14	0.69	27°45′34′′N,111°29′09′′E
LSJ-5	7.44	226.67	93.22	83.83	25.56	—	—	416.61	2.55	0.15	0.84	27°45′34′′N,111°29′09′′E

The numbers represent the mean values (3 replicates).

### The Diversity of Microbial Communities Based on the Metagenomic Datasets

**Figure 2:**
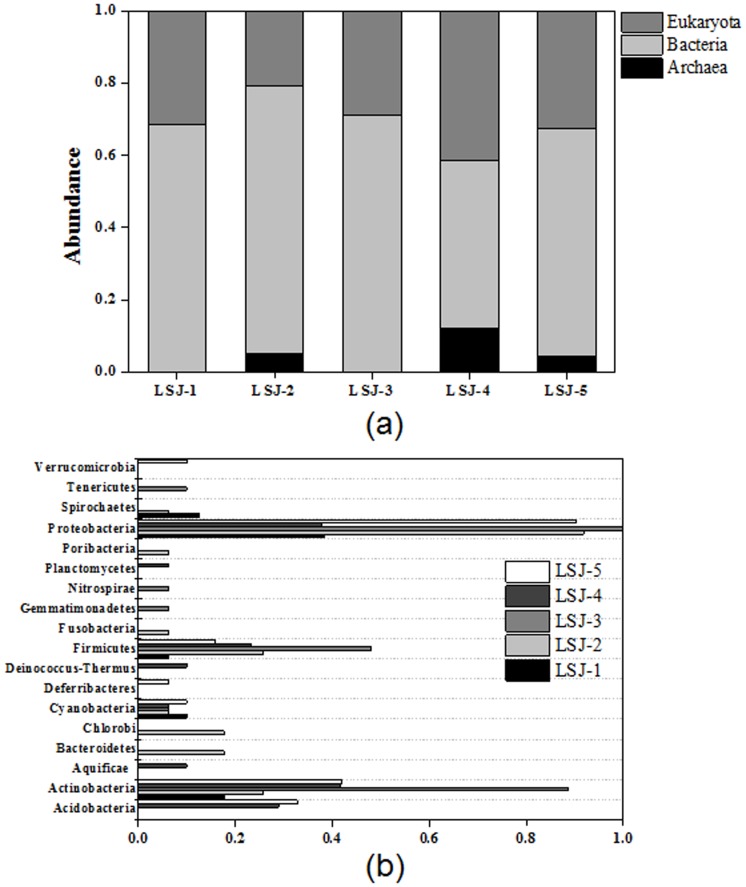
Taxonomic classification of microbial communities in the soil samples at (a) domain level and (b) phylum level. All the information was obtained from metagenomic datasets revealed by Illumina.

After velvet assembly, five metagenomic datasets were uploaded to MG-RAST for taxonomic classification and function annotation. From 16S rRNA gene identification, bacteria play a dominant role among three domains, and account for 68.3%, 74.1%, 71.0%, 46.4%, and 62.9%, among the samples of LSJ-1, LSJ-2, LSJ-3, LSJ-4 and LSJ-5, respectively (**Figure. 2a**). Archaea was not observed in LSJ-1 and LSJ-3. As shown in **Figure. 2b**, *Proteobacteria* had a higher abundance than the others on the phylum level, including six classes (*Betaproteobacteria*, *Gammaproteobacteria*, *Zetaproteobacteria*, *Alphaproteobacteria*, *Epsilonproteobacteria*, and *Deltaproteobacteria*) and unclassified (derived from *Proteobacteria*). *Gammaproteobacteria* was the dominant species among the five soil samples.

The domain analysis among the five samples from the metagenomic datasets indicates that archaea exist universally in the surrounding environment, while archaea is not detected in extreme environments (with extremely high concentrations of As and Sb), such as the area where the LSJ-1 and LSJ-3 samples were collected. *Proteobacteria* is bacteria that can tolerate extremely severe environments and widely exist in the environment. In addition, it can be seen from **Figure. 2b** that *Actinobacteria* is another large group present under the rigorous circumstances, and have a high tolerance to extremely high concentrations of As and Sb as well. *Nitrospirae* and *Gemmatimonadetes* were only found in sample LSJ-3, which had an extremely high concentration of Sb (C_Sb_  = 3923.07 mg/kg). Compared to *Nitrospirae* and *Gemmatimonadetes*, *Chlorobi* and *Bacteroidetes* were only found in the sample LSJ-2 with extremely high concentration of As (C_As_  = 821.23 mg/kg).

### Distribution, Diversity and Abundance of As and Sb Functional Genes

The species transformations of As and Sb is greatly influenced by microorganisms. Thus, it is essential to quantify the abundance of the functional genes related to As and Sb metabolism using Real-time PCR (qPCR). In order to obtain the relative expression of each functional gene, we compared each functional gene's abundance with 16S rRNA bacteria abundance. Rosen et al. [28] and Mukhopadhyay et al. [29], indicate that *arsB* is responsible for As (III) and Sb(III) transfer. While other four genes, such as *arsC*, *arrA*, *arsM* and *aroA* are responsible for As redox and methylation reaction.

As shown in **Figure. S1**, the relative expression of functional genes in different samples showed significant differences. The *arsC* gene had the highest mean relative expression with 1.32×10^−2^ (**Figure. S1. c**),while *arsM* was the lowest with 3.50×10^−4^ (**Figure. S1. e**). Since *arsC* is reported mostly under aerobic conditions, while *arrA* (1.73×10^−3^) is found under anaerobic conditions (**Figure. S1. b**) [16], the higher expression of *arsC* (compared to *arrA*) could indicate that the microbes that reduce As(V) under aerobic conditions may have higher abundance than the anaerobic microbes. The *arsC* and *arrA* gene mediated As(V) reduction process seems to be the most indispensable part of the As biogeochemical cycle. This process can reduce As(V) to As(III), and promote As mobility and bioavailability in the environment [30]. It also contributes to the release of adsorbed As(V) from solid surfaces, such as ferrihydrite and a common soil mineral [31], to the aqueous phase. Relatively high expression was obtained for the *aroA* gene (5.39×10^−3^ (**Figure. S1. a**)), which mediates the As(III) oxidation process. As (III) oxidation is thought to be the microbial detoxification metabolism in various environments that oxidizes toxic As(III) to less toxic As (V). A bioreactor experiment has been carried out to remove As(III) in synthetic groundwater using immobilized As(III)-oxidation bacteria, which were isolated from activated sludge [32]. Meanwhile, As(V) is immobilized and more easily removed than As(III) in aqueous environments [33], [34], especially using FeOOH [35]. The expression of *arsM* was the lowest detected in the five samples. The *arsM* gene can regulate As(III) methylation, which is another detoxification mechanism in microorganisms. Qin et al. [36] showed that *Rhodopseudomonas palustris* can create volatile trimethylarsine (TMAs) as the final product of the As(III) methylation reaction. Since the methylation process of As can volatilize As as an organic form to the atmosphere [37], it is an efficient means to bioremediate As-contaminated soil. Based on the high abundance of redox genes and methylation genes, there is indication that high levels of microbial activities related to As and Sb metabolism exist in the area. The research on Sb metabolism genes and proteins is far more limited than that for As. Until now, only one Sb(V) reduction protein has been identified from *Lershamania*, named LmACR2 [8]. The functional gene *ars*B is related to both Sb(III) and As(III) transport [38]. The relative expression of *arsB* in LSJ-2 was significant higher than in the other four samples, which could be attributed to the high As and Sb concentration (**Figure. S1. d**).

**Figure 3:**
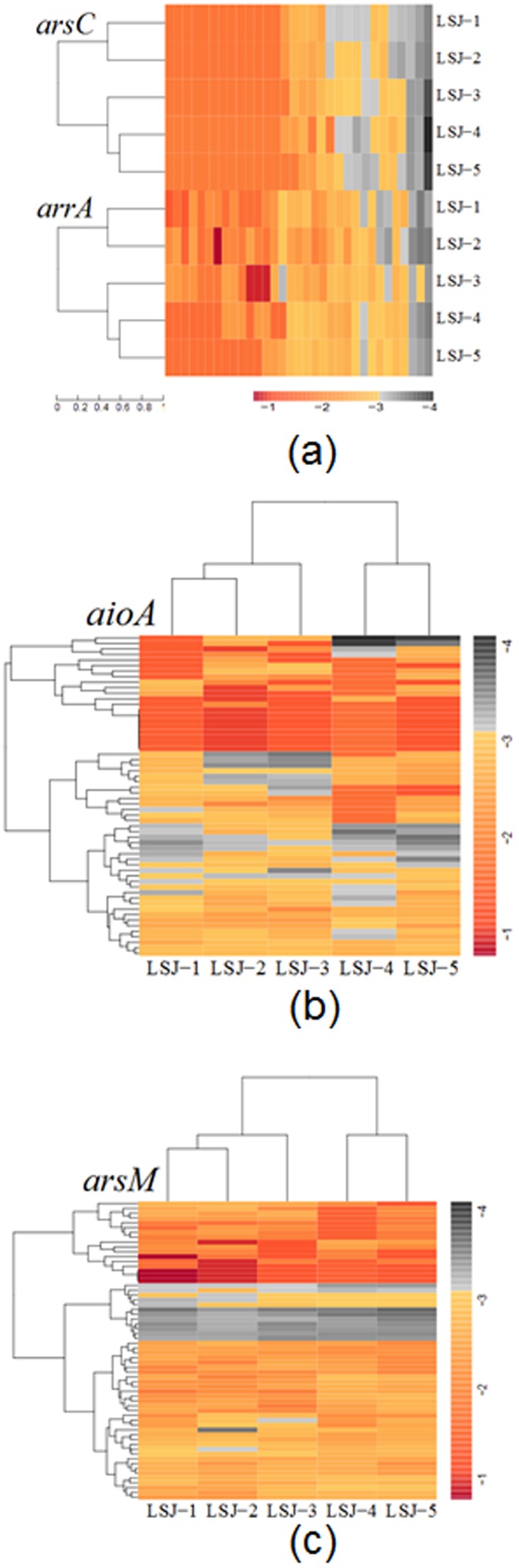
Heat map of log10-transformed proportion of *arsC*-like and *arrA*-like (a), *aioA*-like (b) and *arsM*-like (c) genes distributed in five metagenomic datasets. Phylogenetic analysis used full-length proteins derived from each subdatabase. Complete linkage clustering of five samples was calculated based on the composition and relative abundance of arsenite methylation, arsenite oxidation and arsenate reduction genes. Scales of completely black (−4), pale (−4 to −3), yellow (−3 to −2), orange (−2 to −1), and red(>−1) indicated the abundance of 0%, 0.01–0.1%, 0.1–1%, 1–10%, and >10%, respectively.

It is quite essential to conduct an evaluation of As and Sb functional genes based on the distribution, diversity and abundance of gene levels among the five samples using heatmap analysis. In LSJ-3, *arsC*-like and *arrA*-like genes (**Figure. 3a**) show relatively high distribution, diversity and abundance. Based on analysis of *arsC*-like and *arrA*-like genes, even though the gene distribution was variable, a cluster analysis indicates that LSJ-1 and LSJ-2 share the closest distance and further cluster with LSJ-3, and finally cluster with LSJ-4 and LSJ-5. However, relatively high diversity, distribution and abundance of the *aioA*-like gene is observed in LSJ-2, with the highest concentration of As and relatively high concentration of Sb. Furthermore the cluster analysis of the *aioA*-like gene is the same as *arsC*-like and *arrA*-like genes (**Figure. 3b**). For *arsM*-like genes, it is evident that the LSJ-1 sample shows relatively high diversity when compared with the other four samples (**Figure. 3c**). Based on the analysis above, the diversity, distribution and abundance of all functional genes are relatively high even under extremely high concentrations of As and Sb.

**Figure 4:**
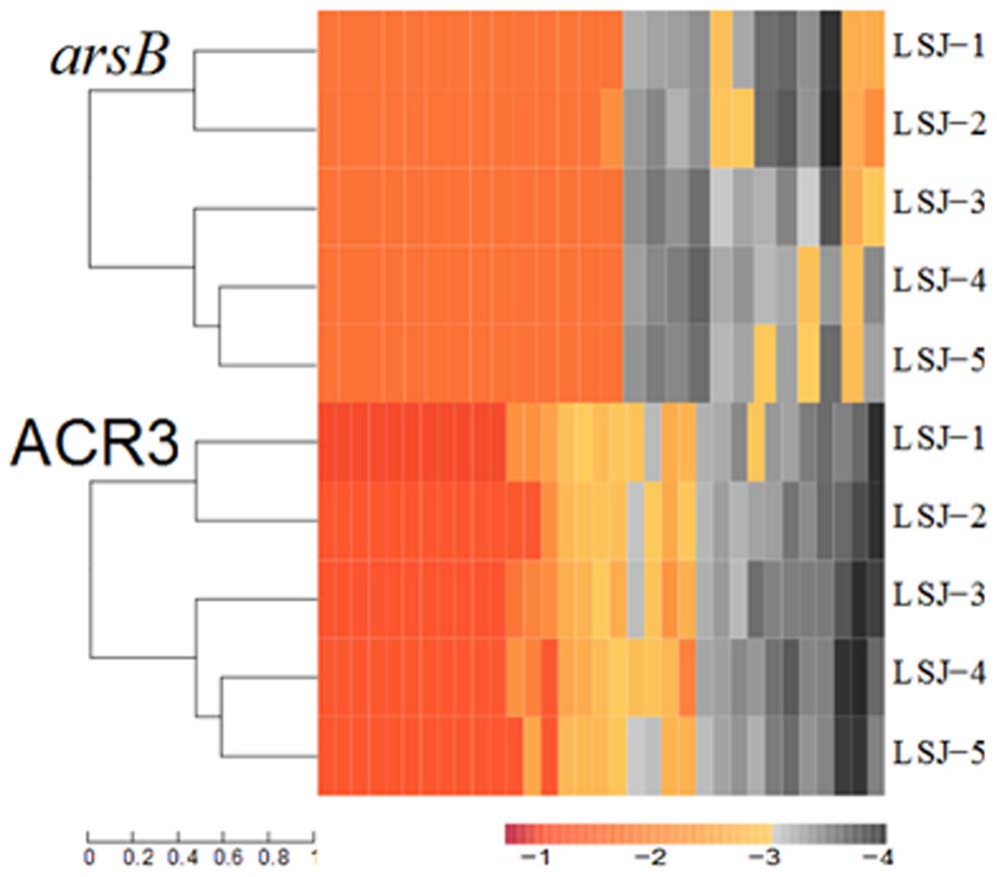
Heat map of log10-transformed proportion of *arsB*-like and ACR3-like genes distributed in five metagenomic datasets. Phylogenetic analysis using full-length proteins derived from each subdatabase. Complete linkage clustering of five samples was calculated based on the composition and relative abundance of arsenite methylation, arsenite oxidation and arsenate reduction genes. Scales of completely black (−4), pale (−4 to −3), yellow (−3 to −2), orange (−2 to −1), and red(>−1) indicated the abundance of 0%, 0.01–0.1%, 0.1–1%, 1–10%, and >10%, respectively.

Two different membrane protein phylogenetic clades, called ArsB and ACR3, can pump out As(III) and Sb(III) from microbial cells [28], [39]. These two proteins were selected to conduct further analysis on their distribution, diversity and abundance among the five soil samples. For ACR3-like genes and *arsB*-like genes, LSJ-2 shows relatively high diversity compared to the other four samples. Even though the gene distribution was variable, cluster analysis of the two samples indicates that LSJ-1 and LSJ-2 share the closest distance and further cluster with LSJ-3, and finally cluster with LSJ-4 and LSJ-5 (**Figure. 4**). The results for the two functional genes could be mainly attributed to the high concentration of As and Sb.

Comparing the results between qPCR and heatmap analysis, high distribution, diversity and abundance of *arsB* was obtained in both analyses. The results of other functional genes in the heatmap were positive with As and Sb concentration, while this tendency was not evidently observed in qPCR. One can mainly attribute the possible discrepancy to the differences between the two methods in analyzing the distribution, diversity and abundance of As- and Sb-related functional genes [40]. Such inconsistent results between qPCR and metagenomic data have also appeared in Yang et al. [41] and Ye et al.'s [42] studies. Ye et al. also claim that metagenomic sequencing was demonstrated to be a better approach than qPCR.

### Statistical Analysis of Microbes, Redox Gene Abundance and Soil Physicochemical Factors

**Figure 5:**
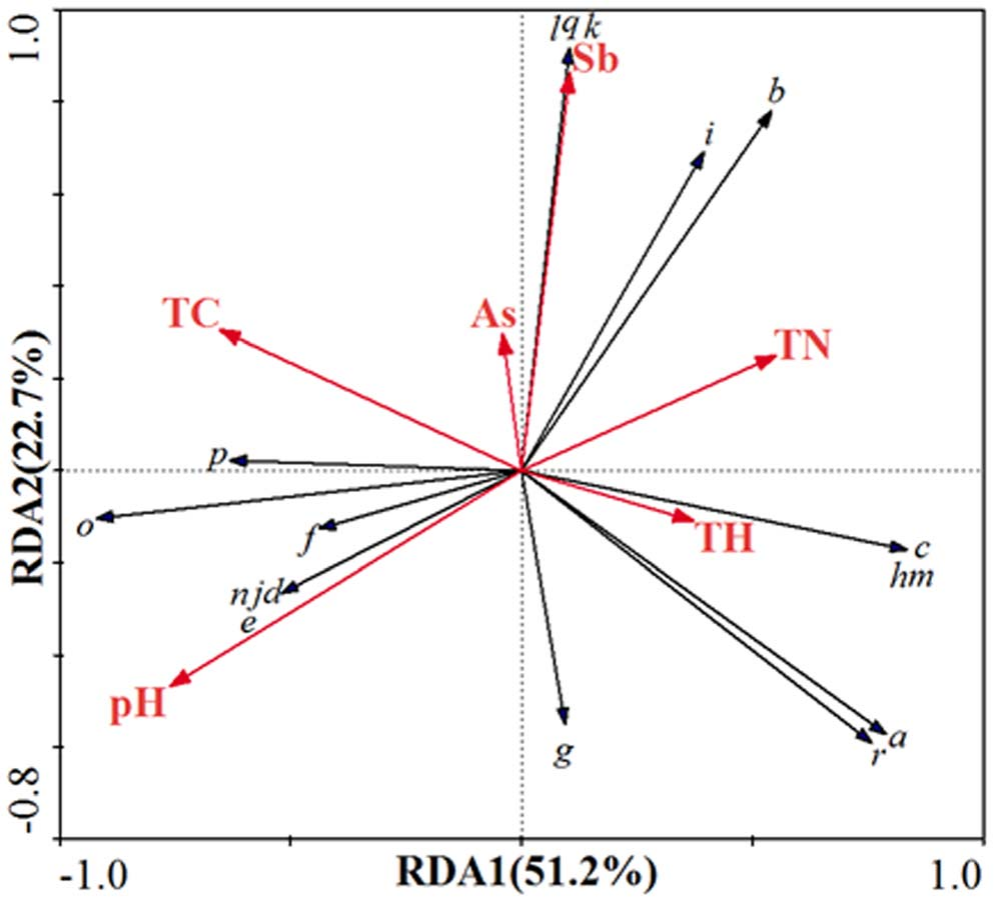
Redundancy analysis (RDA) the relationship between soil physicochemical factors and microbial species under different environmental factors. Axis 1 and axis 2 account for 51.2% and 22.7% of the variance, respectively. a-r indicate different microbes under phylum level: a (Acidobacteria); b (Actinobacteria); c (Aquificae); d(Bacteroidetes); e (Chlorobi); f (Cyanobacteria); g (Deferribacteres); h (Deinococcus-Thermus); i (Firmicutes); j (Fusobacteria); k (Gemmatimonadetes); l (Nitrospirae); m (Planctomycetes); n (Poribacteria); o (Proteobacteria); p(Spirochaetes); q (Tenericutes); r (Verrucomicrobia); TC (total C content); TN (total N content); TH (total H content)

Based on Detrended Correspondence Analysis (DCA) analysis, the length of gradient is less than 3, so a linear model was used in this study [43]. In order to analyze the relationship between microbial species and soil physicochemical factors, RDA analysis was used. As shown in **Figure. 5**, there are multivariate relationships between physicochemical factors and microbial species in the soil samples. The environmental factors, such as pH value and Sb concentration, have great influence on species. Five species (including *Actinobacteria*, *Firmicutes*, *Nitrospirae*, *Tenericutes* and *Gemmatimonadetes*) are positively correlated with As and Sb, indicating that these five species can tolerate high concentrations of As and Sb, and are also dominant species in the environment. Clearly, *Nitrospirae*, *Tenericutes* and *Gemmatimonadetes* are more positively correlated with Sb. This is mainly due to the existence of a detoxification mechanism in these species. Jackson et al. [44] have identified and isolated *Firmicutes* as As(V)-resistant bacteria from As-contaminated woodland soil. Handley et al. [45] have identified *Firmicutes* as a dissimilatory As(V)-reducing prokaryote as well. And based on Joseph et al. [46] report, *Gemmatimonadetes*, as a previously uncultured soil bacterium, has been isolated under laboratory cultivation. Recently, Wang et al. [47] have focused on the study of the inhibition rate of culturable *Actinobacteria* populations under a compound As and Sb pollution system. Integrating these results with our RDA analysis results, we may deduce that *Firmicutes* was more related to As bioremediation, while *Gemmatimonadetes* and *Actinobacteria*, which have been isolated from the soil environment, can serve as a potential bioremediation in both As and Sb contaminated soil. In addition there are another four species highly positively correlated with soil pH, including *Poribacteria*, *Fusobacteria*, *Bacteroidetes* and *Chlorobi*.

**Figure 6:**
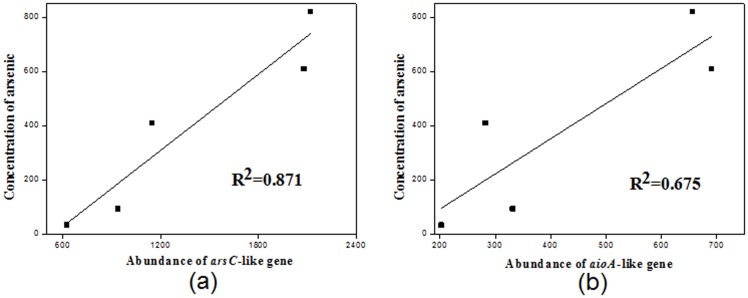
The relationships between the abundance of functional proteins and arsenic concentration across the 5 sites, with x axis showing abundance of functional proteins ((a) indicate *arsC* and (b) indicate *aioA*), y axis showing arsenic concentration. Points are arranged according to the local concentration of arsenic (from low concentration to high concentration, which from 34.1 mg L^−1^ to 821.2 mg L^−1^) corresponding with the abundance of functional proteins

As shown in **Figure. 6**, based on correlation analysis of the relationships between the functional gene abundance and As concentration, a significant linear relation of *arsC*-like genes and As concentration (R^2^ = 0.871) (**Figure. 6a**), as well as *aioA*-like genes and As concentration (R^2^ = 0.675) (**Figure. 6b**), were obtained. The abundance of *arsC*-like and *aioA*-like genes increases with increasing As concentration. *arsC* has been reported to be the dominant gene involved in the intracelluar microbial As detoxification process, which reduces As(III) and then pumps it out to the surrounding environment [48]. *aioA* is also the key gene for As(III) oxidation, which is one of the As detoxification mechanisms as well [49]. With the relatively high R^2^ values for both *arsC* and *aioA*, we strongly propose that the survive of these detected microbes under such a heavily As-contaminated mine field could be attributed to these As detoxification processes.

In summary, using a molecular method (based on metagenomics) and statistical analysis with physicochemical data, this study demonstrates that *Proteobacteria* is the dominant species in high As- and Sb-contaminated environments. Our findings also reveal that As and Sb metabolism genes are diverse, abundant and universal in highly As- and Sb-contaminated environments, and the positive relation between the abundance of As detoxification genes (including *arsC* and *aioA*) and increasing As concentration. This present work promotes our understanding of microbial activities under highly contaminated environments, and opens up an avenue to investigate the relationships between microorganisms and the environment. It could also have implications for us to harness microbes in dealing with environmental issues.

## Supporting Information

Figure S1 **The relative expression of** ***aioA*** **/16S bacteria,** ***arrA*** **/16S bacteria,** ***arsC*** **/16S bacteria,** ***arsB*** **/16S bacteria and** ***arsM*** **/16S bacteria.**
(DOCX)

Table S1 **The accurate locations for the sample collection.**
(DOCX)

Table S2 **The qPCR primer pairs and thermal programs of the functional genes, including** ***arsB*** **,** ***arsC*** **,** ***arrA*** **,** ***arsM*** **and** ***aroA*** **.**
(DOCX)

Table S3 **Accession numbers of arsenic metabolism protein sub-databases involved in** ***arsC*** **-like,** ***arsB*** **-like and ACR3-like genes.**
(DOCX)

Table S4 **Concentrations of arsenic and antimony in environments.**
(DOCX)
