# Supplementary material for: Metagenomic Approach Reveals Variation of Microbes with Arsenic and Antimony Metabolism Genes from Highly Contaminated Soil
Source: PLoS One. 2014 Oct 9;9(10):e108185. doi: 10.1371/journal.pone.0108185 (PMC4191978; doi:10.1371/journal.pone.0108185)
Supplement: Figure S1 — The relative expression of aioA /16S bacteria, arrA /16S bacteria, arsC /16S bacteria, arsB /16S bacteria and arsM /16S bacteria. (DOCX) [file pone.0108185.s001.docx]

(a)

(b)

(c)

(d)

(e)

Figure S1 The relative expression of *aioA***/**16S bacteria(a)*, arrA***/**16S bacteria (b), *arsC***/**16S bacteria (c), *arsB***/**16S bacteria (d) and *arsM***/**16S bacteria (e) among the five soil samples. The R^2^ of the standard curves of all these genes were higher than 0.99.
