# Supplementary material for: Metagenomic Approach Reveals Variation of Microbes with Arsenic and Antimony Metabolism Genes from Highly Contaminated Soil
Source: PLoS One. 2014 Oct 9;9(10):e108185. doi: 10.1371/journal.pone.0108185 (PMC4191978; doi:10.1371/journal.pone.0108185)
Supplement: Table S1 — The accurate locations for the sample collection. (DOCX) [file pone.0108185.s002.docx]

Table S1 The site of samples collection:

| Sample | Site |
| --- | --- |
| LSJ-1 | 27°47’17’’N, 111°27’46’’E |
| LSJ-2 | 27°47’08’’N, 111°29’35’’E |
| LSJ-3 | 27°45’56’’N, 111°29’11’’E |
| LSJ-4 | 27°45’34’’N, 111°29’09’’E |
| LSJ-5 | 27°45’34’’N, 111°29’09’’E |
