# Supplementary material for: Metagenomic Approach Reveals Variation of Microbes with Arsenic and Antimony Metabolism Genes from Highly Contaminated Soil
Source: PLoS One. 2014 Oct 9;9(10):e108185. doi: 10.1371/journal.pone.0108185 (PMC4191978; doi:10.1371/journal.pone.0108185)
Supplement: Table S2 — The qPCR primer pairs and thermal programs of the functional genes, including arsB , arsC , arrA , arsM and aroA . (DOCX) [file pone.0108185.s003.docx]

Table S2 Real-time PCR primer pairs and Thermal programs used in this study

| Target genes | Sequence (5’-3’) of primer pairs | Thermal programs | Reference |
| --- | --- | --- | --- |
| *arsB* | darsB1F:GGTGTGGAACATCGTCTGGAAYGCNAC  darsB1R:CAGGCCGTACACCACCAGRTACATNCC  amlt-42F:TCGCGTAATACGCTGGAGAT  amlt-376R: ACTTTCTCGCCGTCTTCCTT | 30s at 95℃, 40 cycles of 10s at 95℃, 30s at 55℃, and 45s at 72℃ | [1] |
| *arsC* |  | 30s at 95℃, 40 cycles of 5s at 95℃, 30s at 55℃, and 15s at 72℃ | [2] |
| *arrA* | AS1F:CGAAGTTCGTCCCGATHACNTGG  AS1R GGGGTGCGGTCYTTNARYTC  arsMF1: TCYCTCGGCTGCGGCAAYCCVAC  arsMR1: CGWCCGCCWGGCTTWAGYACCCG | 30s at 95℃, 40 cycles of 5s at 95℃, 30s at 55℃, and 45s at 72℃ | [3] |
| *arsM* |  | 30s at 95℃, 40 cycles of 5s at 95℃, 35s at 62℃, and 45s at 72℃ | [4] |
| *aroA* | AroAdeg1F:GTSGGBTGYGGMTAYCABGYCTA  AroAdeg1R:TTGTASGCBGGNCGRTTRTGRAT | 30s at 95℃, 40 cycles of 5s at 95℃, 35s at 62℃, and 45s at 72℃ | [5] |
|  |  |  |  |

**References**

1. Achour AR, Bauda P, Billard P (2007) Diversity of arsenite transporter genes from arsenic-resistant soil bacteria. Res Microbiol 158: 128-137.

2. Sun G (2004) Arsenic contamination and arsenicosis in China. Toxicol Appl Pharmacol 198: 268-271.

3. Song B, Chyun E, Jaffé PR, Ward BB (2009) Molecular methods to detect and monitor dissimilatory arsenate‐respiring bacteria (DARB) in sediments. FEMS microbiology ecology 68: 108-117.

4. Jia Y, Huang H, Zhong M, Wang F-H, Zhang L-M, et al. (2013) Microbial arsenic methylation in soil and rice rhizosphere. Environmental science & technology 47: 3141-3148.

5. Inskeep WP, Macur RE, Hamamura N, Warelow TP, Ward SA, et al. (2007) Detection, diversity and expression of aerobic bacterial arsenite oxidase genes. Environmental microbiology 9: 934-943.
