# Supplementary material for: Metagenomic Approach Reveals Variation of Microbes with Arsenic and Antimony Metabolism Genes from Highly Contaminated Soil
Source: PLoS One. 2014 Oct 9;9(10):e108185. doi: 10.1371/journal.pone.0108185 (PMC4191978; doi:10.1371/journal.pone.0108185)
Supplement: Table S3 — Accession numbers of arsenic metabolism protein sub-databases involved in arsC -like, arsB -like and ACR3-like genes. (DOCX) [file pone.0108185.s004.docx]

Table S3 Accession numbers of arsenic metabolism protein sub-databases involved in *arsC*-like, *arsB*-like and ACR3-like genes

***arsC*-like genes**

| ABO28456.1 | ABO28441.1 | NP_959916.1 | AAA21096.1 |
| --- | --- | --- | --- |
| ABO28455.1 | AAO31600.1 | AFC76436.1 | AAB09628.1 |
| ABO28454.1 | AGW28848.1 | AAS03299.1 | ABO28446.1 |
| ABO28453.1 | CCI75955.1 | ADD77061.1 | ABO28443.1 |
| ABO28452.1 | YP_003520189.1 | AAF89642.1 | AGW28846.1 |
| ABO28445.1 | YP_007974289.1 | AAU03124.1 | ERL66010.1 |
| AFC76434.1 | ADR60568.1 | YP_001405939.1 |  |
| CCE38108.1 | AAR89500.1 | AGC94713.1 |  |

***arsB*-like genes**

| AAU03123.1 | EJZ61645.1 | AFJ15087.1 | EHY00630.1 |
| --- | --- | --- | --- |
| AAO31599.1 | AEJ41862.1 | EIQ68112.1 | EHX95389.1 |
| CCI75954.1 | ADR60567.1 | EIQ50434.1 | EHX88909.1 |
| YP_003520190.1 | EJL12421.1 | EHY16616.1 | EHU72599.1 |
| YP_005930772.1 | ADD77062.1 | EHY12536.1 | EHU82348.1 |
| YP_004721605.1 | ABR80300.1 | EHY04281.1 | AAP32344.1 |

AAP32348.1 AAC69643.1

**ACR3-like genes**

| ACG76371.1 | YP_003066984.1 | YP_001940236.1 | AEA16785.1 |  |
| --- | --- | --- | --- | --- |
| ABF48394.1 | YP_001100235.1 | YP_001514959.1 | ADH07676.1 |  |
| AAM93428.1 | YP_005573076.1 | YP_694398.1 | ACD83638.1 |  |
| EEQ95706.1 | YP_005380238.1 | AFC99719.1 | ABW25645.1 |  |
| CDG51224.1 | YP_007376545.1 | AGC76524.1 | CBH25601.1 |  |
| YP_001211202.1 | YP_003572553.1 | BAF58833.1 | CAX22989.1 |  |
| EFL78225.1 | YP_156506.1 |  |  |  |
| CAL18126.1 | YP_001652104.1 |  |  |  |
| CAL62111.1 | CDG54657.1 |  |  |  |
| Q06598.1 |  |  |  |  |
| EFL81380.1 |  |  |  |  |
| CDG56093.1 |  |  |  |  |
