# Supplementary material for: Metagenomic Approach Reveals Variation of Microbes with Arsenic and Antimony Metabolism Genes from Highly Contaminated Soil
Source: PLoS One. 2014 Oct 9;9(10):e108185. doi: 10.1371/journal.pone.0108185 (PMC4191978; doi:10.1371/journal.pone.0108185)
Supplement: Table S4 — Concentrations of arsenic and antimony in environments. (DOCX) [file pone.0108185.s005.docx]

Table S4 Concentrations of arsenic and antimony in environments

| Location | Arsenic source | Concentration of As | References |
| --- | --- | --- | --- |
| Carniola’s  Oxydizing Wetland, France | Creek | As(III):71±4 mg/L  As(V): 16±1 mg/L | [6] |
| South coast of France | Marine;  sediments | Water: 653 μg/L  Solid : 194 mg/kg | [7] |
| Southern Hong Kong Island  South China Sea (estuarine, onshore and offshore) | Sediments; activated sludge | 1-24 mg/kg | [8] |
| Carnoulès (Gard, France) | Acid  Mine Drainages | 350 mg/L | [9] |
| Slovakia, Central Europe | Soil | 146-540 mg/kg | [10] |
| Macleay River  Floodplain, Australia | Soil | 12.1-22.2 mg/kg | [11] |
| Kapunda and Mount Barker, South Australia | Soil | 121.9±12 mg/L | [12] |
| Spain | Soil | 0.1-25.66 mg/kg | [13] |
| Wupper River, Germany | Soil | 56.1 mg/kg | [14] |

| Location | Antimony source | Concentration of Sb | References |
| --- | --- | --- | --- |
| Lengshuijiang City,  Hunan Province, China | Red soil | 39.29±28.36-528±259 mg/kg | [15] |
| Southern Ontario | Soil | 30.5 mg/kg | [16] |
| Icheonsi, Korea | Soil | 67.48 mg/kg | [17] |
| Macleay River  Floodplain, Australia | Soil | 8.3-16.6 mg/kg | [11] |

**References**

6. Bertin PN, Heinrich-Salmeron A, Pelletier E, Goulhen-Chollet F, Arsène-Ploetze F, et al. (2011) Metabolic diversity among main microorganisms inside an arsenic-rich ecosystem revealed by meta-and proteo-genomics. The ISME journal 5: 1735-1747.

7. Plewniak F, Koechler S, Navet B, Dugat‐Bony É, Bouchez O, et al. (2013) Metagenomic insights into microbial metabolism affecting arsenic dispersion in Mediterranean marine sediments. Molecular ecology 22: 4870-4883.

8. Cai L, Yu K, Yang Y, Chen B-w, Li X-d, et al. (2013) Metagenomic exploration reveals high levels of microbial arsenic metabolism genes in activated sludge and coastal sediments. Applied microbiology and biotechnology 97: 9579-9588.

9. Delavat F, Lett M-C, Lièvremont D (2012) Novel and unexpected bacterial diversity in an arsenic-rich ecosystem revealed by culture-dependent approaches. Biology direct 7: 28.

10. Vaculík M, Jurkovič Ľ, Matejkovič P, Molnárová M, Lux A (2013) Potential risk of arsenic and antimony accumulation by medicinal plants naturally growing on old mining sites. Water, Air, & Soil Pollution 224: 1-16.

11. Wilson SC, Tighe M, Paterson E, Ashley PM (2014) Food crop accumulation and bioavailability assessment for antimony (Sb) compared with arsenic (As) in contaminated soils. Environmental Science and Pollution Research: 1-11.

12. Bolan N, Kunhikrishnan A, Gibbs J (2013) Rhizoreduction of arsenate and chromate in Australian native grass, shrub and tree vegetation. Plant and soil 367: 615-625.

13. Romero-Freire A, Sierra-Aragón M, Ortiz-Bernad I, Martín-Peinado FJ (2014) Toxicity of arsenic in relation to soil properties: implications to regulatory purposes. Journal of Soils and Sediments: 1-12.

14. Frohne T, Rinklebe J, Diaz-Bone RA, Du Laing G (2011) Controlled variation of redox conditions in a floodplain soil: Impact on metal mobilization and biomethylation of arsenic and antimony. Geoderma 160: 414-424.

15. Fu S, Wei CY (2013) Multivariate and spatial analysis of heavy metal sources and variations in a large old antimony mine, China. Journal of Soils and Sediments 13: 106-116.

16. Hale B, Evans L, Lambert R (2012) Effects of cement or lime on Cd, Co, Cu, Ni, Pb, Sb and Zn mobility in field-contaminated and aged soils. Journal of hazardous materials 199: 119-127.

17. Ahmad M, Lee SS, Lim JE, Lee S-E, Cho JS, et al. (2014) Speciation and phytoavailability of lead and antimony in a small arms range soil amended with mussel shell, cow bone and biochar: EXAFS spectroscopy and chemical extractions. Chemosphere 95: 433-441.
